# Trimethylamine N‐Oxide Mitigates Perioperative Neurocognitive Disorders via ANXA1 Nuclear Translocation and M2 Microglial Polarization in the Hippocampus

**DOI:** 10.1111/cns.70558

**Published:** 2025-08-10

**Authors:** Mengxin Que, Li Luo, Xuan Wang, Shiyong Li, Qian Xia, Xing Li, Ailin Luo, Gaofeng Zhan

**Affiliations:** ^1^ Department of Anesthesiology and Pain Medicine, Hubei Key Laboratory of Geriatric Anesthesia and Perioperative Brain Health, and Wuhan Clinical Research Center for Geriatric Anesthesia, Tongji Hospital, Tongji Medical College Huazhong University of Science and Technology Wuhan China

## Abstract

**Aims:**

This study investigates whether trimethylamine N‐oxide (TMAO) mitigates perioperative neurocognitive disorders (PND) by modulating Annexin A1 (ANXA1) and microglial polarization, thereby reducing neuroinflammation in the hippocampus.

**Methods:**

A murine PND model was established via tibial fracture surgery under sevoflurane anesthesia. Mice were pretreated with TMAO (1.2, 12, or 120 mg/kg) for 21 days. Cognitive function was assessed using Y‐maze and fear conditioning tests. Hippocampal ANXA1 expression, microglial polarization (M1/M2 phenotypes), and cytokine levels (TNF‐α, IL‐1β, TGF‐β) were analyzed.

**Results:**

TMAO administration (12 mg/kg) significantly improved cognitive performance. Mechanistically, TMAO upregulated ANXA1 expression, facilitating its nuclear translocation in microglia and shifting their polarization from pro‐inflammatory M1 phenotype to anti‐inflammatory M2 phenotype. This transition consequently suppressed pro‐inflammatory cytokines (TNF‐α and IL‐1β) while elevating TGF‐β. Additionally, TMAO attenuated microglial activation and associated neuroinflammatory morphological alterations.

**Conclusion:**

Physiological concentrations of TMAO confer neuroprotection by augmenting ANXA1‐mediated resolution of neuroinflammation, supporting its therapeutic potential for preventing PND.

## Graphical abstract

This schematic depicts the proposed mechanism by which TMAO alleviates PND by modulating neuroinflammation in the hippocampus. Anesthesia and surgery disrupt the balance between pro‐inflammatory (e.g., IL‐1β, TNF‐α) and anti‐inflammatory cytokines (e.g., TGF‐β), promoting M1 microglial polarization and cognitive impairment. TMAO pretreatment upregulates ANXA1, facilitating its nuclear translocation and shifting microglia toward the anti‐inflammatory M2 phenotype. This transition reduces pro‐inflammatory cytokines, suppresses microglial activation, and improves cognitive function. The diagram highlights key mediators (TMAO, ANXA1, TGF‐β) and cellular responses (microglial polarization, cytokine shifts) underlying TMAO's neuroprotective effects in PND.

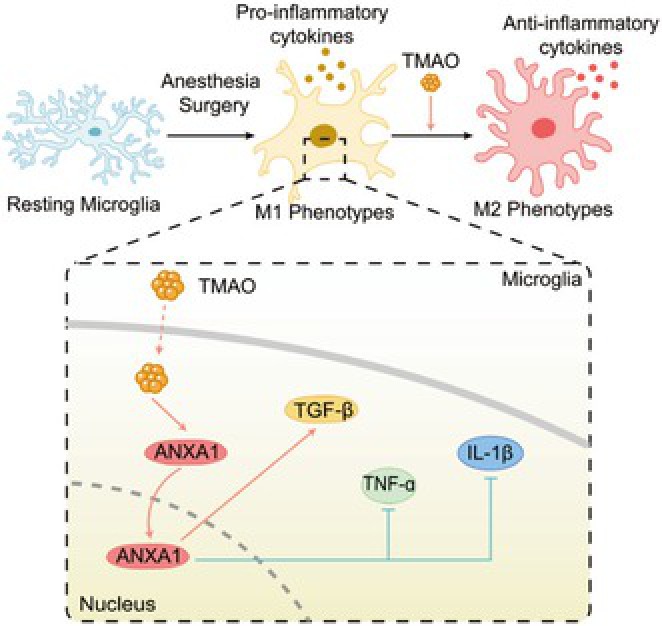

## 1 Introduction

Perioperative neurocognitive disorders (PND) are neurological complications frequently observed in elderly patients following anesthesia and surgery. They present substantial clinical challenges due to their adverse effects on postoperative recovery, prolonged hospitalization, and increased risk of long‐term disability. Contributing factors include advanced age [1], preoperative anxiety [2], perioperative sleep disturbances [3], and perioperative sedation/analgesia management strategies [4]. Despite extensive research, effective therapeutic interventions remain limited, largely due to the incomplete understanding of the pathophysiological mechanisms involved. Neuroinflammation in the hippocampus has emerged as a key factor contributing to PND, with immune cells such as microglia playing a central role, which can adopt pro‐inflammatory (M1) or anti‐inflammatory (M2) phenotypes. Although M1‐type microglia exacerbate inflammation, M2‐type microglia help counteract these responses, offering a pathway to potentially reduce the neuroinflammation associated with PND.

Annexin A1 (ANXA1) is a Ca^2+^‐regulated phospholipid‐binding protein. It was well‐documented as a glucocorticoid‐regulated anti‐inflammatory protein [5]. ANXA1 is known to mediate the resolution of inflammation by modulating the activity of immune cells and influencing cellular signaling pathways. Recent studies suggest that ANXA1 may influence microglial polarization, promoting the transition from a pro‐inflammatory to an anti‐inflammatory state [6], which is particularly relevant in neuroinflammatory conditions like PND. Ac2‐26, an N‐terminal peptide of ANXA1, can trigger the formyl peptide receptor‐like 1 and play a role in ameliorating microglial activity, reducing neuroinflammation in the hippocampus of Alzheimer's disease mice, and alleviating cognitive deficits [7]. ANXA1 also regulates the polarization of microglia/macrophages and protects against cerebral ischemia–reperfusion injury through the FPR2/ALX‐dependent AMPK‐mTOR pathway [6]. The cellular localization of ANXA1, including its cytoplasmic translocation, plasma membrane translocation, and nuclear translocation [8], are also believed to impact its role in neuronal apoptosis [9, 10] and microglia‐induced inflammation regulation [11]. Underscoring the need to explore how ANXA1 expression dynamics can contribute to neuroinflammatory modulation in PND.

Various physiological channels—such as the neuroendocrine system, the autonomic nervous system, neuroimmune pathways, and molecules produced by gut microbes—serve as a bridge between the gut and the brain [12]. The gut microbiota has the ability to metabolize choline and L‐carnitine from foods such as red meat, eggs, and fish, leading to the production of trimethylamine (TMA). This TMA, produced by the gut microbiota, is absorbed into the bloodstream and transported to the liver, where it is converted to TMAO [13] through the action of hepatic flavin monooxygenases 3 and subsequently released into circulation. TMAO has been detected in cerebrospinal fluid in both clinical and animal studies, indicating that it can cross the blood–brain barrier [14, 15]. Research has also shown that the FMO3 enzyme exists in the brain, suggesting that TMAO may be synthesized from TMA within the brain [16]. Although the relationship between TMAO and cognitive decline remains incompletely characterized, evidence indicates dose‐dependent neuromodulatory effects: physiologically relevant TMAO concentrations exhibit neuroprotective properties [17]. However, when elevated beyond physiologically relevant concentrations, TMAO promotes oxidative stress and neuroinflammation in the brain [18, 19]. Oxidative stress and neuroinflammation are recognized factors contributing to PND [20, 21]. High circulating levels of TMAO may lead to the activation of microglia, an increase in reactive astrocytes, and subsequently result in neuroinflammation [22]. At physiological concentrations, TMAO acts as a protector in maintaining blood brain barrier integrity by regulating ANXA1, a tight junction regulator [17]. Whether physiological concentrations of TMAO decrease neuroinflammation through the regulation of ANXA1 remains unclear.

This work provides insight into a novel pathway including TMAO, ANXA1, and microglia polarization, which are implicated in PND. We found that a noticeable decrease in ANXA1 and increased nuclear translocation can induce microgliosis and microglia polarization in the hippocampus of PND mice, whereas TMAO treatment improves these results. This research elucidates the underlying mechanisms and identifies potential targets for addressing PND.

## 2 Materials and Methods

### 2.1 Animals

Male C57BL/6 mice (6–8 months old, weighing 25–30 g) were purchased from the Laboratory Animal Center of Tongji Medical College. All animals were housed under a 12‐h light/dark cycle, 22°C–25°C temperature, 45%–65% humidity, with ad libitum access to food and water. All experimental procedures were approved by the Institutional Animal Care and Use Committee (IACUC) and conducted in compliance with the ethical standards for animal research. Male C57BL/6 mice were divided randomly into five experimental groups: (1) Control, (2) anesthesia + surgery (AS), (3) AS + 1.2 mg/kg TMAO, (4) AS + 12 mg/kg TMAO, and (5) AS + 120 mg/kg TMAO. Trimethylamine N‐oxide (TMAO) was dissolved in distilled water and given to the mice through drinking water for 21 consecutive days before undergoing anesthesia and surgery. Group sizes were preestablished based on data from previously published studies and our earlier experiments. Exact sample sizes (*n*) are specified in the figure legends. Measures were taken to minimize animal suffering and reduce the number of animals used, adhering to ethical standards.

### 2.2 Establishment of PND Model

Based on both published studies and our prior research [23], we induced PND in mice using tibial fracture and intramedullary fixation under sevoflurane anesthesia (2% isoflurane, 98% oxygen). After disinfecting the skin, the left tibia was exposed, stabilized with a 0.3 mm pin, and then fractured. The incision was sutured using a 5‐0 Vicryl thread, and bupivacaine (0.5%, 1 mg/kg) was administered locally to manage pain. Lidocaine cream was applied twice daily for 1–3 days after surgery to alleviate incision pain. Throughout the procedure, a heating blanket maintained the body temperature at 37°C ± 0.5°C.

### 2.3 Behaviors Assessment

Cognitive and locomotor functions were evaluated through behavioral assessments. These tests were conducted in a dark, quiet, soundproof room with a controlled, comfortable temperature. Behavioral data were recorded using a tracking system (Zhongshi Technology, Beijing, China). Each test was carried out by two individuals who were blinded to the group assignments.

#### 2.3.1 Open Field Test (OFT)

Mice were placed in an open field apparatus (50 cm × 50 cm × 50 cm) for 5 min to assess spontaneous locomotor activity. Total distance traveled and time spent in the center zone were recorded using an automated tracking system.

#### 2.3.2 Y‐Maze Test

The Y‐maze apparatus comprised three uniform compartments arranged in a 120° equiangular configuration, with each chamber measuring 30 cm (length) × 15 cm (height) × 6 cm (width). These compartments were designated as the initial, other, and novel zones. Experimental procedures consisted of two distinct phases separated by a 4‐h intermission. During the acquisition trial, access to the unfamiliar zone was physically obstructed while the subject was positioned at the entrance of the initial compartment for a 3‐min exploration period. The subsequent retention trial maintained identical parameters except for the removal of barriers preventing access to the previously unavailable zone. Behavioral parameters including frequency of entries and time spent in the novel environment were systematically quantified. Following each testing session, all contact surfaces underwent thorough cleaning with a 5% acetic acid solution to remove residual scents from prior trials.

#### 2.3.3 Fear Conditioning Test (FCT)

The behavioral apparatus consisted of a rectangular polycarbonate chamber (25 × 25 × 30 cm) with a stainless steel grid floor connected to a programmable shock generator. Contextual and cued fear memory assessments were conducted across two sequential phases separated by 24 h. During the conditioning session, subjects were exposed to a 30‐s auditory tone (5 kHz, 75 dB) co‐terminating with a 2‐s mild footshock (0.5 mA) delivered through the floor grid. This tone‐shock pairing was repeated three times with 60‐s intertrial intervals.

Twenty‐four hours post‐conditioning, memory retrieval was evaluated in two distinct contexts: (1) Contextual fear testing occurred in the original conditioning chamber (unaltered environment), whereas (2) Cued fear testing was performed in a modified chamber with altered geometric patterns, olfactory cues (1% vanilla extract), and illuminated lighting conditions. During cued testing, the auditory stimulus was presented continuously for 180 s without footshock reinforcement.

Freezing behavior (complete immobility excluding respiration) was quantified using automated video tracking software (EthoVision XT 15). Parameters included latency to first freezing episode, cumulative freezing duration, and freezing bout frequency. All sessions were video recorded under suitable illumination to permit behavioral analysis. Environmental parameters (ambient noise < 50 dB, humidity 45%–55%, temperature 22°C ± 1°C) were strictly controlled. Chamber surfaces were sanitized with 70% ethanol between trials to eliminate contextual odor residues.

### 2.4 Western Blot Analysis

Total proteins were extracted using prechilled RIPA lysis buffer (Beyotime Biotechnology, China). Following centrifugation (12,000 ×*g*, 15 min, 4°C), the supernatant was collected and subjected to heat denaturation at 95°C for 5 min. Protein separation was performed via sodium dodecyl sulfate‐polyacrylamide gel electrophoresis (SDS‐PAGE); after which the resolved proteins were electrophoretically transferred onto PVDF membranes (Roche, Basel, Switzerland). Membranes were blocked with 5% nonfat milk for 1 h at ambient temperature, followed by overnight incubation with primary antibodies at 4°C. HRP‐conjugated anti‐rabbit and anti‐mouse IgG secondary antibodies were employed for immunodetection. Protein signals were visualized using an enhanced chemiluminescence substrate (Abbkine, USA) and quantified via densitometric analysis (Image Lab software). β‐Tubulin served as the internal reference for normalization of target protein expression levels. Detailed antibody specifications are provided in Table 1.

**TABLE 1:** All primary and secondary antibodies used in this study.

Antibody	Species	Source	Identifier	IF	WB
ANXA1	Rabbit	Abcam	ab214486	1:200	1:1000
CD206	Rabbit	Abcolonal	A21014		1:1000
CD86	Rabbit	Abcolonal	A21198		1:1000
CD68	Rabbit	HUABIO	HA722285	1:200	
CD32	Rabbit	Abcolonal	A1388		1:1000
Histone H3	Rabbit	Abcolonal	A22146		1:1000
Iba1	Goat	Abcam	ab5076	1:200	
β‐tubulin	Mouse	Proteintech	10094‐1‐AP		1:1000
Anti‐rabbit IgG HPR	Goat	Proteintech	SA00001‐2		1:1000
Anti‐mouse IgG HRP	Goat	Proteintech	SA00001‐1		1:1000

### 2.5 Immunofluorescence (IF)

Mice were transcardially perfused with PBS followed by 4% paraformaldehyde 24 h after anesthesia and surgery. The brains were completely excised, fixed in 4% paraformaldehyde, and initially dehydrated in 20% sucrose until they sank to the bottom. They were then transferred to 30% sucrose and processed following the same protocol. Using a freezing microtome (Leica CM1900, Wetzlar, Germany), the brains were sliced into 10‐μm sections. Brain tissues were permeabilized with 0.3% Triton X‐100 for 20 min, blocked with 5% donkey serum for 1 h, and incubated overnight with primary antibodies against ANXA1 and CD68. The following day, samples were incubated with fluorescently labeled secondary antibodies (Alexa Fluor [488/594]) for 1 h. DAPI (4′,6‐diamidino‐2‐phenylindole) was used to stain nuclei. Images were captured using a confocal microscope (Leica, Germany). Fluorescently labeled samples were imaged with a Zeiss confocal microscope (Zeiss, LSM800, Oberkochen, Germany). Quantitative analysis of immunofluorescence staining images was performed by a blinded investigator using ImageJ software. Specifically, we opened the original image file with ImageJ software, circled Iba1 microglia using the rectangle tool, and then obtained the total intensity of green light‐labeled ANXA1 and CD68 using the measurement function. All primary and secondary antibodies are listed in Table 1.

### 2.6 Statistical Analysis

Data are presented as mean ± standard error of the mean. To evaluate the normality of continuous variables, we performed both graphical assessments (Q‐Q plots) and formal statistical testing (Shapiro–Wilk tests) to assess potential deviations from normality, including skewness and kurtosis. Data were analyzed using one‐way ANOVA (Tukey's post hoc test) when comparing more than two groups under a single factor, and two‐way ANOVA (Tukey's post hoc test) when more than two groups and two factors were compared. Comparisons between two independent groups were performed using unpaired two‐tailed Student's *t*‐tests. For non‐normally distributed data, nonparametric tests (e.g., the Mann–Whitney *U* and Kruskal–Wallis tests) were utilized. Statistical significance was set at *p* < 0.05, with symbols representing significance levels as follows: *p < 0.05, **p < 0.01, ***p < 0.001, ****p < 0.001; NS indicates not significant. Data analysis was conducted using GraphPad Prism software, version 9.0.

## 3 Results

### 3.1 TMAO Pretreatment Ameliorates Cognitive Dysfunction Following Anesthesia and Surgery

**FIGURE 1:**
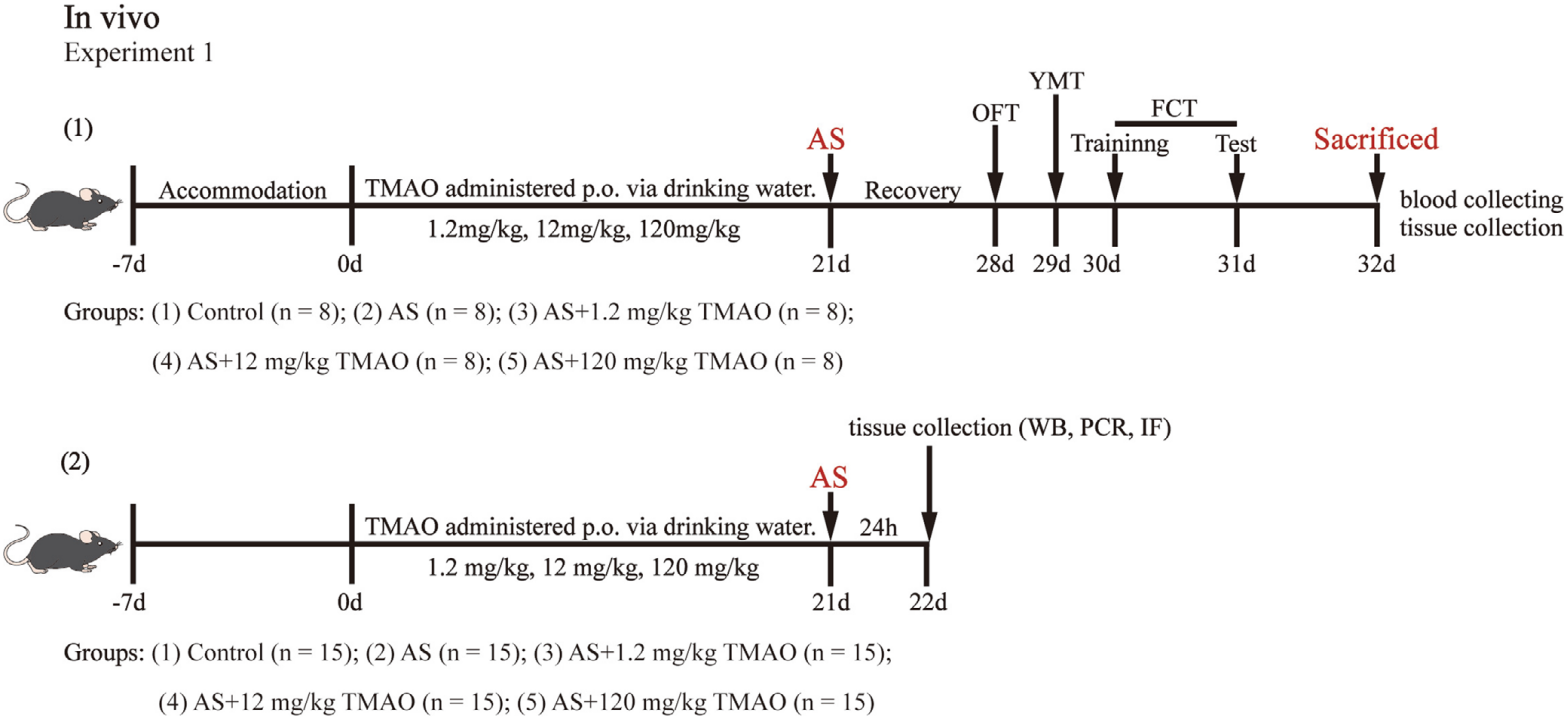
Experimental timeline and group design. Mice were pretreated with TMAO (1.2, 12, or 120 mg/kg) for 21 days, followed by tibial fracture surgery under sevoflurane anesthesia. Behavioral tests (open field test, Y‐maze test, and fear conditioning test) were conducted on postoperative Day 7. Tissues were collected for biochemical and histological analyses at designated time points.

**FIGURE 2:**
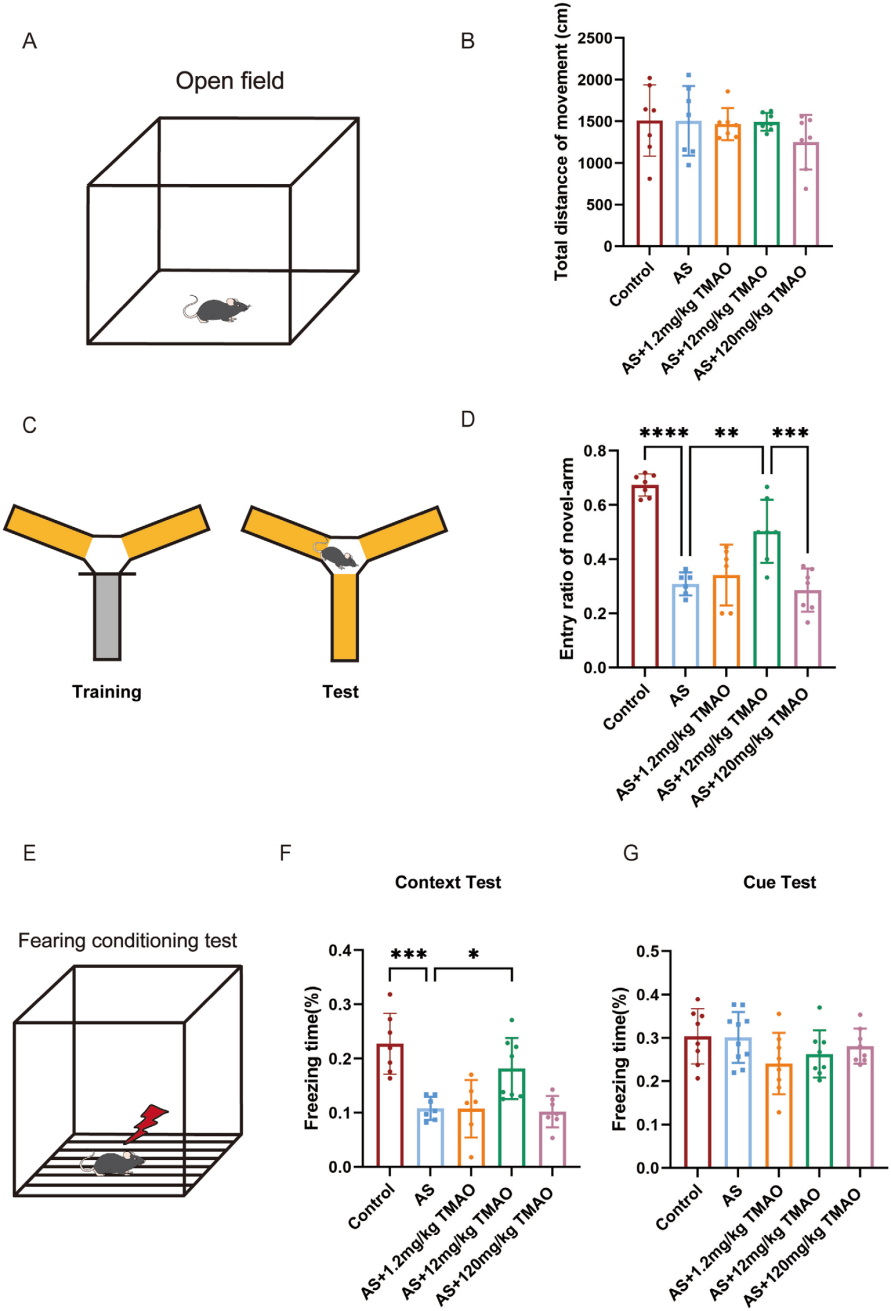
TMAO pretreatment improved cognitive function in postoperative mice. Behavioral assessments of cognitive and locomotor functions in control, AS, and TMAO‐pretreated groups. (A, B) Open field test (OFT) showing no significant differences in total distance traveled. (C, D) Y‐maze test (YMT) demonstrating improved spatial memory in TMAO‐pretreated groups, particularly at 12 mg/kg. (E–G) Fear conditioning test (FCT) showing increased freezing time in TMAO‐pretreated mice, indicating enhanced memory retention. Data are presented as mean ± SEM. Statistical analysis was performed using two‐way ANOVA with Tukey's post hoc test. *p < 0.05; **p < 0.01; ***p < 0.001.

To determine if TMAO pretreatment can alleviate cognitive dysfunction induced by anesthesia and surgery, we randomly assigned mice into five groups: Control, AS, AS+1.2 mg/kg TMAO, AS+12 mg/kg TMAO, AS+120 mg/kg TMAO. Behavioral tests were conducted until the seventh day post‐anesthesia and surgery (Figure 1). Initially, the OFT was conducted to assess the consistency of locomotor activity across groups. The findings revealed no significant difference in the total distance covered (Figure 2A,B). Subsequently, we used the Y‐maze and FCTs to evaluate spatial learning and memory function, respectively. The Y‐maze results showed that anesthesia and surgery reduced the number of times mice entered the new arms, an effect that was reversed by 12 mg/kg TMAO pretreatment (Figure 2C,D). Similarly, in the FCT, postoperative mice showed a significant decrease in freezing time, whereas those in the 12 mg/kg TMAO pretreatment group maintained prolonged freezing behavior (Figure 2E–G). In summary, 12 mg/kg TMAO pretreatment ameliorates cognitive dysfunction following anesthesia and surgery.

### 3.2 TMAO Pretreatment Modulates Microglial Morphologies and Microglia Phenotypes After Anesthesia and Surgery

**FIGURE 3:**
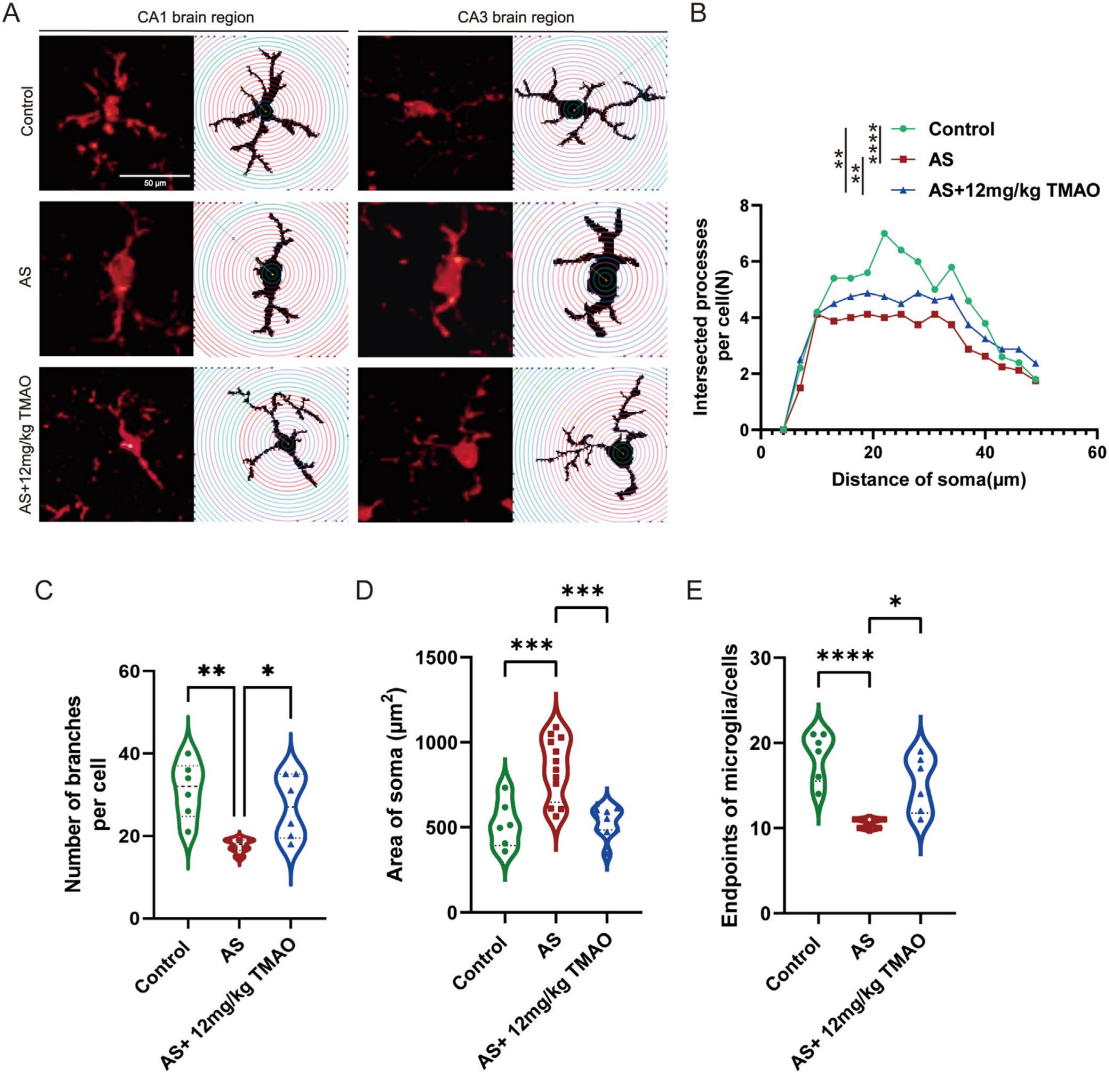
TMAO pretreatment attenuated microglial morphological changes induced by anesthesia and surgery. Immunofluorescence staining of Iba1+ microglia in the hippocampus. (A, B) Sholl analysis showing reduced microglial branching and process length in the AS group, which was reversed by TMAO pretreatment. (C–E) Morphometric quantification of microglial branches and junctions. Data are presented as mean ± SEM. Statistical analysis was performed using one‐way ANOVA with Tukey's post hoc test. *p < 0.05; **p < 0.01 vs. AS group.

To investigate the effect of TMAO pretreatment on dynamic anesthesia and surgery induced changes in microglial morphology. As indicated in the schematic diagram, the animals were sacrificed at 24 h. The Iba1 immunostaining (red) and the skeleton and sholl analyses reported enlarged cell bodies and thickened and shortened microglial processes in the hippocampus after anesthesia and surgery (Figure 3A–E). Morphometric quantification demonstrated that after anesthesia and surgery, the branches and junctions of microglia were significantly reduced when compared with those of the TMAO pretreatment mice (Figure 3C, *p* < 0.05). In physiological conditions, the total process length of normal microglia was significantly longer than that of anesthesia and surgery‐induced microglia. Under TMAO stimulation, however, the process length of microglia in mice after anesthesia and surgery was notably longer when compared with that of the mice after anesthesia and surgery. The sholl analysis showed that, compared with the microglial morphology in mice after anesthesia and surgery, less ramified microglia were evident in normal mice and TMAO pretreatment mice, and that anesthesia and surgery stimulation notably shortened the branches and transformed them into an “activated state” (Figure 3A,B).

### 3.3 TMAO Pretreatment Promotes the Switch of Hippocampal Microglia From the M1 to the M2 Type After Anesthesia and Surgery

**FIGURE 4:**
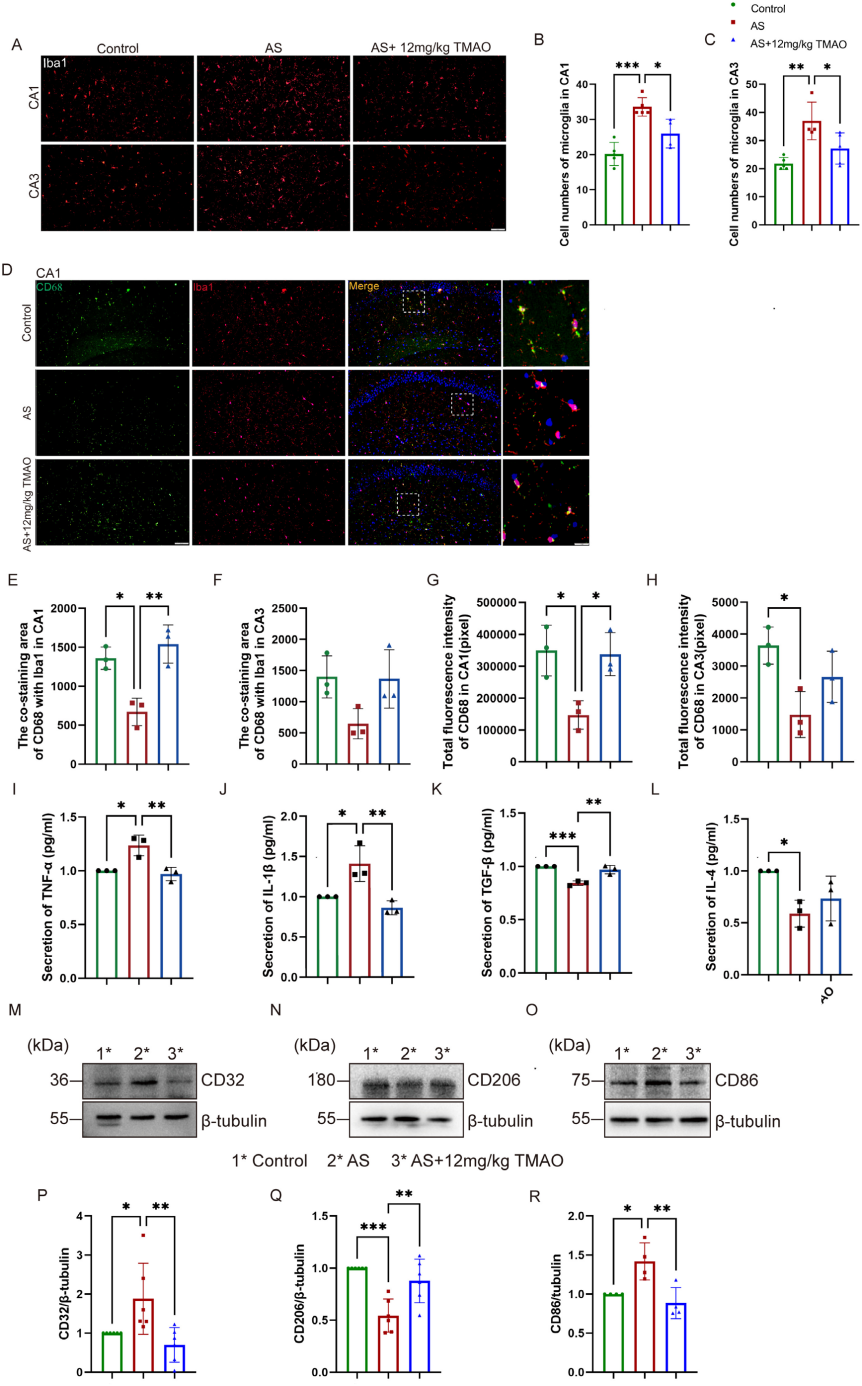
TMAO pretreatment promoted microglial polarization from M1 to M2 phenotype. (A–C) Confocal microscopy images of hippocampal microglia with Iba1 (red) and quantitative analysis of microglial cell numbers. (D–H) Quantitative analysis of M2 marker (CD68) expression and total fluorescence intensity. (I–L) ELISA results showed reduced pro‐inflammatory cytokines (TNF‐α, IL‐1β) and increased anti‐inflammatory cytokine (TGF‐β) levels in TMAO‐pretreated groups. (M–O) Western blot analysis of M1 (CD32) and M2 (CD206, CD86) markers. (P–R) Quantification results of Western blot showed that the expression of CD32 and CD86 was increased in the AS group compared with the control and the AS+12 mg/kg TMAO group. Statistical significance was determined by one‐way ANOVA followed by Tukey's post hoc test. *p < 0.05, **p < 0.01, ***p < 0.001.

Microglial polarization is a well‐established response to neuroinflammation. Numerous markers may be expressed in microglia, playing a role in regulating their phenotypic changes during morphological transitions [24, 25]. We further investigated whether TMAO could influence M1/M2 microglial polarization in the PND model; we employed confocal microscopy to analyze the several microglial phenotype markers. As shown in Figure 4A–C, there was a marked increase in the number of microglia in the hippocampal CA1 and CA3 regions following 24 h after anesthesia and surgery.

Compared to anesthesia and surgery mice, the normal physiological condition mice have higher levels of the co‐staining area of the M2 marker CD68 with Iba1 in the hippocampal CA1, and total fluorescence intensity (Figure 4D–H). Furthermore, enzyme‐linked immunosorbent assay results demonstrated that TMAO pretreatment reversed the increase in pro‐inflammatory cytokines, including TNF‐α and IL‐1β (*p* < 0.01) (Figure 4I,J), and countered the decrease in anti‐inflammatory cytokines TGF‐β (*P* < 0.01) but not differences in IL‐4 (Figure 4K,L). To test this hypothesis, we also conducted western blot to evaluate the expression of microglial polarization markers. Anesthesia and surgery significantly elevated exprthe ession of the M1 macrophage marker CD32 (*p* < 0.01) (Figure 4M). Concurrently, M2 markers, such as CD206 and CD68, were reduced after anesthesia and surgery (*p* < 0.01) (Figure 4N,O). Our findings suggest that TMAO pretreatment facilitates the shift of hippocampal microglia from the M1 to the M2 phenotype following anesthesia and surgery.

### 3.4 TMAO Pretreatment Enhances Hippocampal ANXA1 Expression Following Anesthesia and Surgery

**FIGURE 5:**
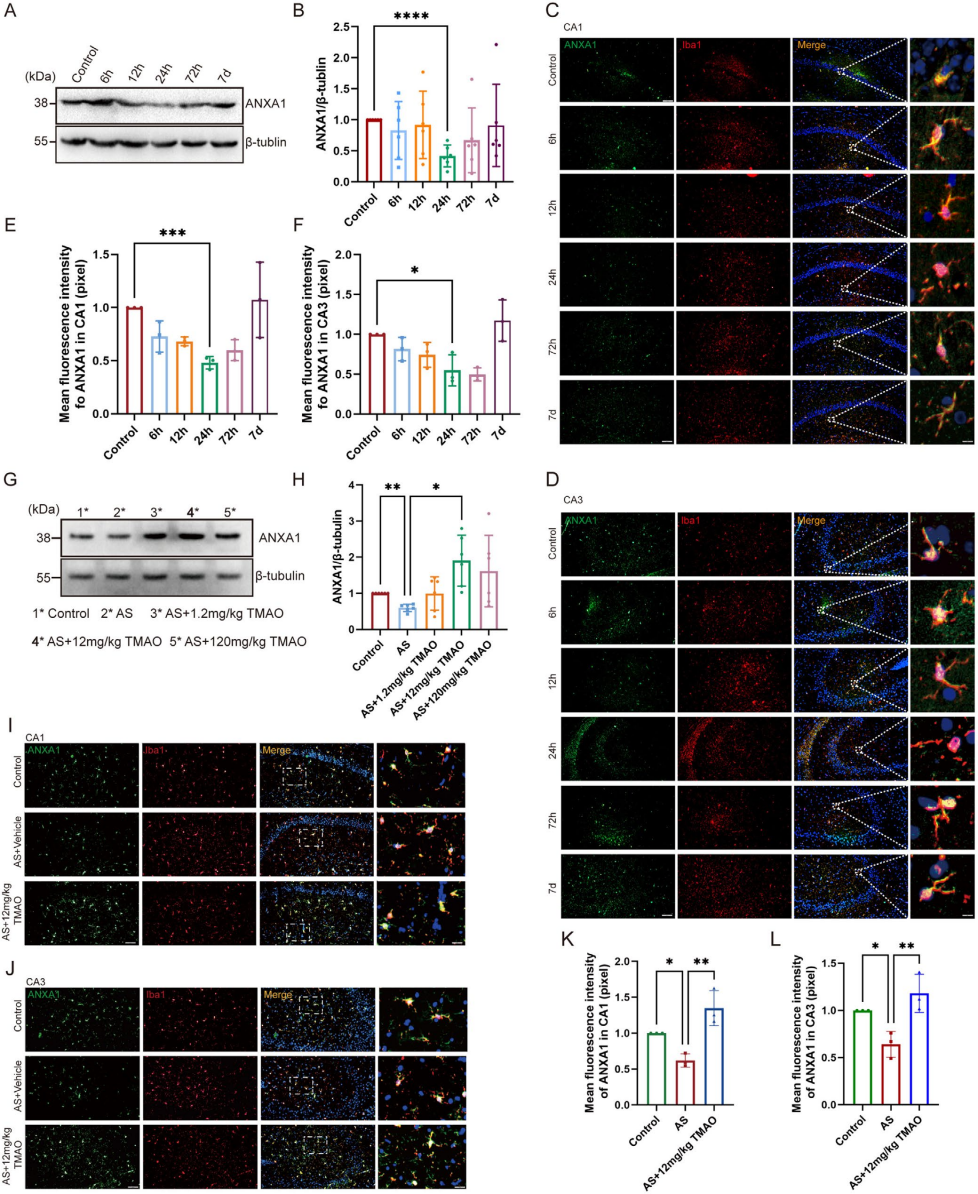
TMAO pretreatment enhanced hippocampal ANXA1 expression. (A, B) Time‐dependent changes in hippocampal ANXA1 expression post‐anesthesia and surgery, with the lowest levels observed at 24 h. (C–F) Immunofluorescence analysis of ANXA1 expression in hippocampal CA1 and CA3 regions. (G, H) Western blot showing restored ANXA1 expression in TMAO‐pretreated groups. (I–L) Confocal images of ANXA1 expression in microglia. Data are presented as mean ± SEM. Statistical analysis was performed using paired t‐tests for pairwise comparisons (B, E, F, H) and one‐way ANOVA with Tukey's post hoc test (H, K, L). *p < 0.05, **p < 0.01, ***p < 0.001.

ANXA1 is a critical regulator of the neuroinflammatory response, but its contribution to PND and the role it plays in the efficacy of TMAO remain unclear. The levels of ANXA1 in the hippocampus of mice showed a trend of initially decreasing and then increasing across six time points after anesthesia and surgery: 6 h, 12 h, 24 h, 72 h, and 7 days. Notably, ANXA1 levels reached their lowest point at the 24‐h mark post‐surgery (Figure 5A,B). Immunofluorescence analysis shows the lowest expression level at 24 h post‐surgery in the hippocampal CA1 (*p* < 0.001) and CA3 (*p* < 0.05) (Figure 5C–F). We believe this pattern may be attributed to the dynamic process of neuroinflammation and resolution, where the initial decrease in ANXA1 is related to the acute inflammatory response, followed by an increase as the inflammation begins to resolve and the brain initiates recovery processes [26]. And then, we investigated whether TMAO pretreatment could exert its effects by regulating ANXA1. Our findings revealed that TMAO pretreatment restored the diminished ANXA1 expression caused by anesthesia and surgery at the 24‐h post‐surgery (Figure 5G,H). Furthermore, immunofluorescence analysis demonstrated that TMAO pretreatment significantly increased ANXA1 expression in microglia within the hippocampal CA1 and CA3 regions post‐anesthesia and surgery (Figure 5I–L). Collectively, these results suggest that TMAO pretreatment may alleviate cognitive dysfunction after anesthesia and surgery by upregulating ANXA1 expression.

### 3.5 TMAO Pretreatment Increased the Nuclear Translocation of ANXA1 Following Anesthesia and Surgery

**FIGURE 6:**
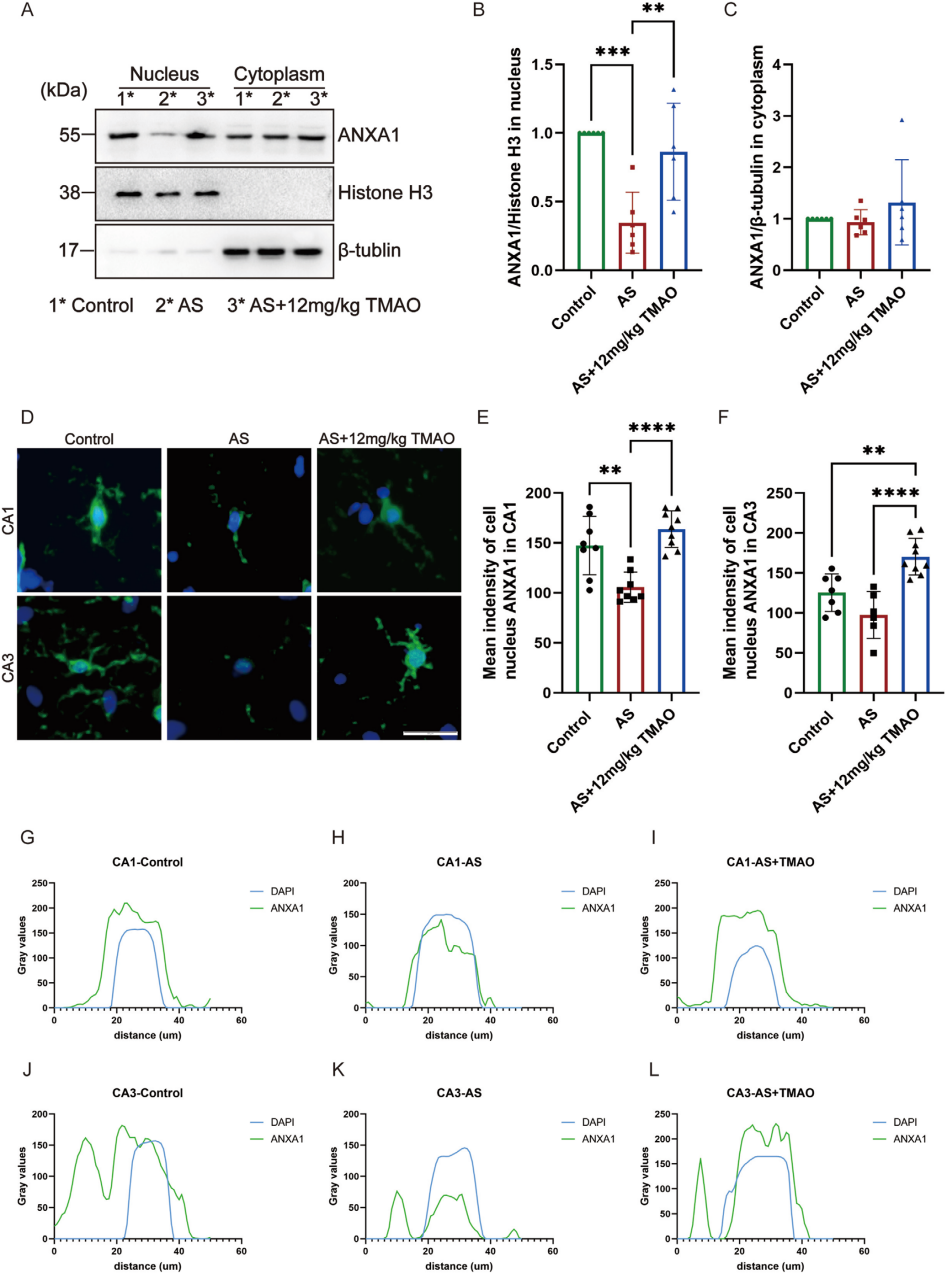
TMAO pretreatment increased nuclear translocation of ANXA1 in hippocampal microglia. (A–C) Western blot results showing ANXA1 nuclear translocation in hippocampal microglia. (D–F) Immunofluorescence analysis of nuclear ANXA1 expression in CA1 and CA3 regions. (G–L) Co‐localization analysis of ANXA1 (green) and DAPI (blue) in microglia, showing increased nuclear translocation in TMAO‐pretreated groups. Data are presented as mean ± SEM. Statistical significance was determined by one‐way ANOVA followed by Tukey's post hoc test. *p < 0.05, **p < 0.01, ***p < 0.001.

ANXA1 is a key protein involved in the resolution of inflammation. In our study, we found that pretreatment with TMAO before anesthesia and surgery significantly promoted the shift of hippocampal microglia from the M1 to the M2 phenotype. The M2 type of microglia is known for its anti‐inflammatory properties, which are essential for cognitive recovery following surgical procedures. The shift was associated with an increase in the nuclear translocation of ANXA1 (Figure 6A–C). Furthermore, we conducted immunofluorescence to test ANXA1 expression in the cell nucleus of both CA1 and CA3. The result shows a decreased expression of nuclear ANXA1 after anesthesia and surgery, compared with TMAO pretreatment (Figure 6D–F). Co‐localization analysis between the nuclear marker DAPI and ANXA1 was performed in hippocampal microglia (Figure 6G–L). Quantitative fluorescence profiling demonstrated that in mice subjected to anesthesia and surgery, the relative intensity of DAPI signals (blue channel) at identical pixel coordinates significantly surpassed that of ANXA1 signals (green channel), compared to TMAO‐pretreated surgical counterparts. This spatial distribution disparity (DAPI > ANXA1) indicates diminished nuclear expression of ANXA1. These findings suggest that TMAO may play a protective role by facilitating the switch from M1 to M2 microglia, thereby facilitating improvement of anesthesia and surgery‐induced cognitive disorders. The observed increase in ANXA1's nuclear translocation further underscores TMAO's potential as a therapeutic agent in mitigating neuroinflammation and preserving cognitive function after surgery.

## 4 Discussion

The relationship between trimethylamine N‐oxide (TMAO) and PND has garnered increasing attention due to its dual role in neuroinflammation and cognitive regulation. The neurological impact of TMAO exhibits a dose‐dependent dichotomy. Although high‐dose TMAO demonstrates well‐established neurotoxic effects [19, 27], a number of studies regard TMAO as a potentially beneficial agent, which is a stabilizer of the protein‐folded state and nucleic acids [28, 29, 30]. Specifically, TMAO has been implicated in maintaining blood–brain barrier integrity, reducing oxidative stress, and modulating neuroinflammatory responses [17]. These properties position TMAO as a potential therapeutic candidate for mitigating PND. To date, the neuroprotective mechanisms of physiological doses remain poorly understood. In this study, we explored the protective effects of TMAO pretreatment in a murine model of PND, focusing on its ability to regulate ANXA1 expression and microglial polarization.

To investigate the dose‐dependent effects, mice were administered three distinct TMAO concentrations for 21 days prior to sacrifice. In nature, mammalian animals, TMAO can play a role as a nondeleterious osmolyte and cellular protectants in renal cortex and medulla, enhancing excretion of some toxic substances [31]. However, excessive TMAO can induce oxidative stress and endothelial dysfunction, impairing renal excretory capacity [32, 33]. This adaptive response may explain the better cognitive performance of mice observed in the intermediate‐dose group.

Neuroinflammation has been considered the main pathological factor contributing to PND development [21, 34]. Emerging evidence suggested that suppression of neuroinflammation could attenuate cognitive deficits caused by laparotomy surgery and left tibial fracture surgery under sevoflurane anesthesia [35, 36, 37]. ANXA1 is an endogenous mediator which mimics the effects of glucocorticoids [38], plays an important role in the immune system, including the regulation of microglial activation, maintenance of endothelial integrity, and modulation of leukocyte trafficking [26, 39]. Supplementation with a biomimetic peptide derived from ANXA1 in elderly mice following anesthesia and surgery alleviates neuroinflammation and reduces microglial activation, thereby improving perioperative cognitive function [26]. Meanwhile, administration of the ANXA1 N‐terminal pharmacophore peptide (Ac2‐26) during cerebral ischemia–reperfusion injury promoted the polarization of microglia/macrophages toward the anti‐inflammatory M2 phenotype in the ischemic penumbra [6].

In studies of ischemic stroke, nuclear accumulation of ANXA1 in neurons and microglia was associated with neuronal apoptosis and microglial polarization [40, 41]. However, contrasting evidence from research on high‐fat diet (HFD)/streptozotocin (STZ)‐induced kidney injury suggests that increased nuclear expression of ANXA1 in renal tubular epithelial cells facilitates its interaction with p65, thereby inhibiting NF‐κB activation and attenuating downstream inflammatory responses [42]. These findings suggest the role of ANXA1 nuclear translocation appears to be context‐dependent and may vary due to differences in experimental models and time points of observation. We demonstrated that the anesthesia and surgery caused a time‐dependent expression of ANXA1 and M1 innate immunity genes (CD68) in microglia. Meanwhile, TMAO treatment increased the expression of ANXA1 in microglia, promoted the switch of hippocampal microglia from the M1 to the M2 type, and decreased inflammatory factors of the hippocampus at 24 h after anesthesia and surgery. Furthermore, TMAO pretreatment increased ANXA1 nuclear translocation; we hypothesize that TMAO‐induced nuclear enrichment of ANXA1 in the anesthesia/surgery group inhibits specific pro‐inflammatory signaling pathways, ultimately reducing neuroinflammation and improving cognitive outcomes. The protective role of ANXA1 nuclear translocation in this context differs from its reported pro‐inflammatory function in ischemic stroke models, suggesting that the functional consequences of ANXA1 nuclear translocation are highly dependent on the underlying pathophysiological conditions. Further studies are needed to elucidate the precise molecular mechanisms governing ANXA1 localization and its downstream effects in different neuroinflammatory settings.

Despite these promising findings, several limitations should be considered. First, our study focused primarily on hippocampal microglia, yet PND involves a complex interplay between multiple brain regions and other cell types, including astrocytes and peripheral immune cells. Future studies should explore the systemic effects of TMAO on neuroinflammation and cognitive function beyond the hippocampus. Second, whereas we demonstrated that TMAO modulates ANXA1 expression and nuclear translocation, the precise signaling pathways involved remain unclear. ANXA1 is known to interact with key inflammatory mediators, such as p65/IL‐1β [43]. In the context of the TMAO pretreatment PND model, further studies should aim to identify the signaling pathways regulated by ANXA1 nuclear translocation using the co‐immunoprecipitation method. Finally, although our findings suggest that TMAO exerts dose‐dependent effects, the optimal therapeutic window for TMAO administration remains undetermined. More comprehensive dose–response studies, including long‐term safety evaluations, are necessary before considering clinical translation.

These findings uncover a previously unrecognized role of TMAO in modulating microglia ANXA1 function and support an essential conceptual advance that appropriate intake of TMAO can play a positive role in reducing neuroinflammation following anesthesia and surgery. Our findings suggest that TMAO, at optimal concentrations, can attenuate neuroinflammation and improve cognitive outcomes, highlighting its potential as a novel intervention for PND prevention.

## Author Contributions

Mengxin Que collected and analyzed the data. Li Luo and Xuan Wang oversaw the biological sample analysis. Shiyong Li was responsible for figure and table preparation. Qian Xia and Xing Li provided essential resources to facilitate data collection and analysis. Gaofeng Zhan and Ailin Luo conceptualized and designed the study. All authors contributed to manuscript editing and approved the final version.

## Disclosure

Institutional Review Board Statement: All experiments were strictly following the National Institute of Health Guide for the Care and Use of Laboratory Animals and approved by the Laboratory Animal Welfare and Ethics Committee of Tongji Hospital, Tongji Medical College, Huazhong University of Science and Technology (approval number: TJH‐202303043).

## Conflicts of Interest

The authors declare no conflicts of interest.

## Supporting information


**Data S1:** cns70558‐sup‐0001‐DataS1.jpg.

## Data Availability

The data supporting our study's findings are presented in the article. The corresponding author can provide the date upon reasonable request.
