# Supplementary figures and images for: Trimethylamine N‐Oxide Mitigates Perioperative Neurocognitive Disorders via ANXA1 Nuclear Translocation and M2 Microglial Polarization in the Hippocampus
Source: CNS Neurosci Ther. 2025 Aug 10;31(8):e70558. doi: 10.1111/cns.70558 (PMC12336294; doi:10.1111/cns.70558)

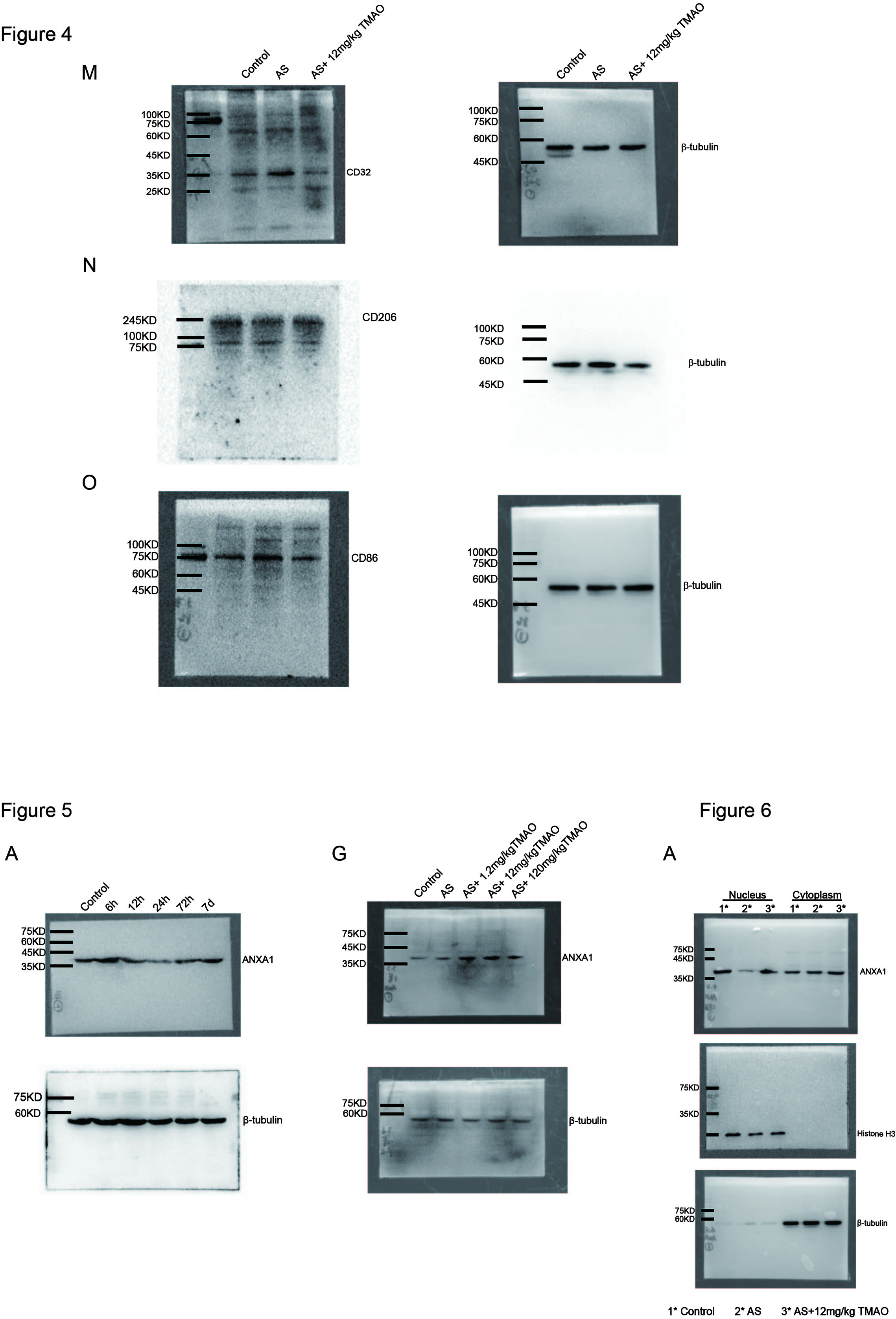

Supplement: Supplementary file 1 — Data S1: cns70558‐sup‐0001‐DataS1.jpg. [file CNS-31-e70558-s001.jpg]
